# Effects of Whey Protein Hydrolysate Ingestion on Postprandial Aminoacidemia Compared with a Free Amino Acid Mixture in Young Men

**DOI:** 10.3390/nu10040507

**Published:** 2018-04-19

**Authors:** Kyosuke Nakayama, Chiaki Sanbongi, Shuji Ikegami

**Affiliations:** Food Science & Technology Research Laboratories, Meiji Co., Ltd., 1-29-1 Nanakuni, Hachiouji, Tokyo 192-0919, Japan; chiaki.sanbongi@meiji.com (C.S.); shuuji.ikegami@meiji.com (S.I.)

**Keywords:** whey protein hydrolysate, aminoacidemia, leucine, essential amino acids, muscle protein synthesis

## Abstract

To stimulate muscle protein synthesis, it is important to increase the plasma levels of essential amino acids (EAA), especially leucine, by ingesting proteins. Protein hydrolysate ingestion can induce postprandial hyperaminoacidemia; however, it is unclear whether protein hydrolysate is associated with higher levels of aminoacidemia compared with a free amino acid mixture when both are ingested orally. We assessed the effects of whey protein hydrolysate (WPH) ingestion on postprandial aminoacidemia, especially plasma leucine levels, compared to ingestion of a free amino acid mixture. This study was an open-label, randomized, 4 × 4 Latin square design. After 12–15 h of fasting, 11 healthy young men ingested the WPH (3.3, 5.0, or 7.5 g of protein) or the EAA mixture (2.5 g). Blood samples were collected before ingestion and at time points from 10 to 120 min after ingestion, and amino acids, insulin, glucose and insulin-like growth factor-1 (IGF-1) concentrations in plasma were measured. Even though the EAA mixture and 5.0 g of the WPH contained similar amounts of EAA and leucine, the WPH was associated with significantly higher plasma EAA and leucine levels. These results suggest that the WPH can induce a higher level of aminoacidemia compared with a free amino acid mixture when both are ingested orally.

## 1. Introduction

Muscle mass can be considered one of the most important components of the human body because it generates force and movement and is a major site of metabolism. The amount of muscle mass is regulated by the net balance between muscle protein synthesis (MPS) and breakdown [1,2]. Ingestion of protein or amino acids can increase the net muscle protein balance, primarily because of the increase in MPS, with a lesser contribution from the decrease in muscle protein breakdown [3,4]. Elevation of the concentrations of plasma essential amino acids (EAA), especially that of leucine, may be one of the key factors for stimulating MPS, as a positive correlation has been reported between plasma EAA and leucine levels and MPS [5,6]. Additionally, a rapid acute rise in postprandial circulating EAA or leucine levels following consumption of protein-rich food has been associated with increases in MPS [7,8,9]. Our previous rodent study demonstrated that hyperaminoacidemia following consumption of acidified milk was associated with a greater increase in MPS compared with skim milk, although the milk drinks had the same types and quantities of protein [7]. In addition, West et al. [8] demonstrated that increases in MPS after whey protein ingestion were smaller when the whey protein was ingested in small pulses that mimicked a more slowly digested protein, and those pulses were associated with a lower degree of aminoacidemia. Furthermore, Koopman et al. [9] suggested that ingestion of casein hydrolysate to yield more pronounced aminoacidemia was more effective for elevating the rate of MPS compared with ingestion of intact casein. Given these results, postprandial plasma EAA or leucine levels, especially peak EAA or leucine concentrations, could be indicators for increases in MPS.

The rates of protein digestion and absorption appear to be important factors for achieving rapid postprandial aminoacidemia according to studies comparing whey protein and casein, known as “fast” and “slow” proteins respectively [5,10,11]. Hydrolysis of protein is an effective strategy to accelerate its digestion and absorption rates [9] and induce hyperaminoacidemia [12]. Free amino acids are also absorbed rapidly because they are absorbed directly through amino acid transporters without being digested, but previous animal studies have shown that after intestinal infusion, amino acids from protein hydrolysate compared with free amino acids appeared in the portal or peripheral blood more rapidly [13,14].

We previously demonstrated that the ingestion of whey protein hydrolysate (WPH) was associated with a greater increase in MPS compared with an amino acid mixture in rodents [15]. Although these results may have been caused by a faster and greater development of aminoacidemia after WPH ingestion, it remains unclear whether a protein hydrolysate, including the WPH we used in the previous study, induces greater aminoacidemia compared with a free amino acid mixture when ingested orally.

In the present study, we investigated the effects of ingestion of the WPH on postprandial aminoacidemia, especially plasma leucine levels, compared to the effects of an amino acid mixture. We also regard the present study as a preliminary study to estimate the minimum effective dose of the WPH for stimulating MPS through postprandial aminoacidemia; we will then use that dose in human clinical trials to investigate muscle protein turnover. Therefore, as a control, we used the same EAA mixture that Cuthbertson et al. [16] and Tipton et al. [17] used for their human studies to investigate the relationship of EAA ingestion with MPS. Cuthbertson et al. [16] demonstrated that MPS was stimulated in a dose-dependent fashion. We used their minimum effective dose (2.5 g of EAA) as the control dose and compared it to 3 doses (3.3, 5.0 and 7.5 g of protein) of the WPH. We hypothesized that ingestion of the medium dose (5.0 g) of the WPH, which contained similar amounts of EAA and leucine to 2.5 g of the EAA mixture, may cause equal or greater postprandial aminoacidemia than that of the EAA mixture.

## 2. Materials and Methods

### 2.1. Subjects

Twelve healthy young men were recruited as study volunteers. All subjects gave written informed consent prior to participation in this study and then underwent physical and medical examinations, urinalysis and blood tests including complete blood count with differential, liver and kidney function tests, fasting blood glucose, and hepatitis B and C screening. The procedures in this study were approved by the Institutional Review Board of the Chiyoda Paramedical Care Clinic (Tokyo, Japan) (ethic approval code: MIJ16C3) and the Meiji Institutional Review Board (Tokyo, Japan) (ethic approval code: 76), and conducted in accordance with ethical principles laid down by the Declaration of Helsinki and Ethical Guidelines for Medical and Health Research Involving Human Subjects (Ministry of Health, Labour and Welfare, Japan). The trial was registered in the UMIN Clinical Trials Registry (UMIN-CTR No. UMIN 000021108).

### 2.2. Experimental Design

This study was an open-label, randomized, 4 × 4 Latin square design, conducted at the Chiyoda Paramedical Care Clinic (Tokyo, Japan). Each subject participated in 4 experiments designed to measure the concentrations of amino acids in plasma before and following ingestion of 3.3, 5.0 or 7.5 g of WPH or 2.5 g of the EAA mixture. The Latin square design for four treatments is shown in Table 1. The repeat experiments were separated by washout periods of at least 6 days.

### 2.3. Experimental Procedures

The subjects refrained from ingesting alcohol and from smoking on the day before each experiment, and all consumed the same meal (612 kcal) 12–15 h before the start of each experiment. Thereafter, the subjects were allowed to ingest only water until the experiments started.

On the day of each experiment, the subjects underwent physical and medical examinations (body weight, blood pressure, pulse rate and physical conditions), and the baseline venous blood samples were drawn from the antecubital vein (*t* = 0 min). The subjects were randomized to ingest 1 of the 4 test solutions, and venous blood samples were drawn 10, 20, 30, 45, 60, 90 and 120 min after ingestion of the test solution.

### 2.4. Test Solutions

We used 4 test solutions: 3 different doses (3.3, 5.0 and 7.5 g of protein) of the WPH (Meiji Co., Ltd., Tokyo, Japan) and 2.5 g of the EAA mixture (Kyowa Hakko Bio Co., Ltd., Tokyo, Japan), each dissolved in 200 mL of water. The amino acid composition of the EAA mixture matched that in previous muscle protein turnover studies [16,17]. Table 2 lists the EAA content and other nutrients of the test solutions. The WPH had an average peptide length of 3.50, which was determined by a o-phthaldialdehyde reaction method [18]. The WPH contained 2.1 mg of free amino acids per 1.0 g of the WPH.

### 2.5. Blood Analyses

Whole blood samples were collected in vacutainers containing sodium fluoride and the disodium salt of ethylenediaminetetraacetic acid (EDTA)-Na_2_ for plasma glucose analyses, and were collected in vacutainers containing only EDTA-Na_2_ for other plasma analyses. After collection, the blood samples were centrifuged at 3000 rpm (730 g) for 5 min at 4 °C, and plasma samples were stored at −80 °C until assayed. Plasma free amino acids were measured by high-performance liquid chromatography-mass spectrometry (ACQUITY TQD, Waters Corporation, Milford, MA, USA) with pre-column 6-aminoquinolyl-*N*-hydroxysuccinimidyl carbamate derivatization [19].

Plasma insulin concentrations were measured using a chemiluminescence immunoassay (Architect Insulin, Abbott Japan Co., Ltd., Tokyo, Japan), glucose concentrations were measured using enzymatic methods (Iatoro LQ GLU, Unitica Ltd., Osaka, Japan), and insulin-like growth factor-1 (IGF-1) levels were determined using an immunoradiometric assay (IGF-1 (Somatomedin-c) IRMA Daiichi, Fujirebio Inc., Tokyo, Japan).

### 2.6. Statistics

All values are expressed as mean ± standard error of the mean (SEM). The effects of period, subject and sequence were analyzed with one-factor analysis of variance (ANOVA) using the data of plasma concentration changes from baseline at each time point. Plasma concentration changes from baseline were analyzed using a repeated measures two-factor ANOVA (4 × 8; treatment, time). Differences between the means of the EAA mixture and of each dose of the WPH at the same time point were assessed by using paired-sample *t* tests with Holm–Bonferroni corrections when significant interactions between treatment and time were found. The data of the area under the curves (AUCs) for plasma concentration changes were analyzed using a repeated measures one-factor ANOVA with a Dunnett’s post hoc test for comparison of each dose of the WPH with the EAA mixture. Holm–Bonferroni corrections were calculated by using Microsoft Excel (Microsoft Corp., Redmond, WA, USA) and the other analyses were performed by using SPSS for Windows, version 23 (IBM Japan, Ltd., Tokyo, Japan). The overall significance was set at *p* < 0.05, and the Holm–Bonferroni-adjusted significance to compare the first-ranked (smallest) *p*-value was set at *p* < 0.05/21.

## 3. Results

### 3.1. Subject Characteristics

One subject (Sequence 2) dropped out during the study and was excluded from analysis. The characteristics for the 11 subjects who completed the study were as follows: age, 24.5 ± 0.8 year; height, 173.4 ± 2.2 cm; body mass, 60.9 ± 1.8 kg; and body mass index, 20.5 ± 0.5 kg/m^2^.

### 3.2. Plasma Amino Acids

**Figure 1:**
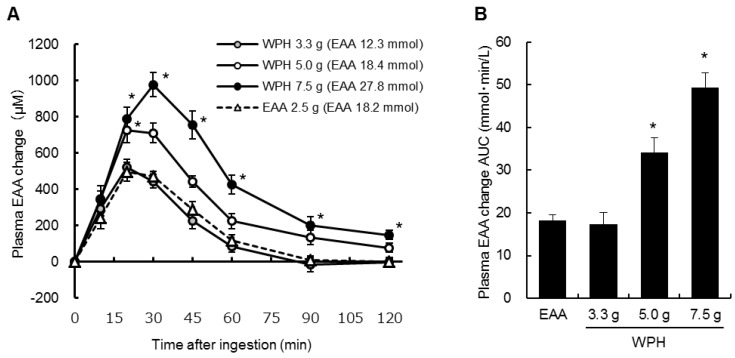
Plasma EAA concentration (**A**) changes from baseline and (**B**) AUCs. Values are mean ± standard error of the mean. * Significantly different from EAA, *p* < 0.05. AUC: area under the curve; WPH: whey protein hydrolysate; EAA: essential amino acids.

Figure 1A,B shows respectively, the changes from baseline and the AUCs of the plasma EAA concentrations (including arginine, which is a semi-essential amino acid). Significant treatment × time interactions were observed in the EAA concentration changes from baseline (*p* < 0.001). The EAA concentration changes at 20–120 min after ingestion of 7.5 g of the WPH and at 20 min after ingestion of 5.0 g of the WPH were significantly higher than the changes after ingestion of the EAA mixture at the same time points. The plasma EAA concentration AUCs after ingestion of 5.0 and 7.5 g of the WPH were also significantly higher than that of the EAA mixture. There were no significant differences between the ingestion of the EAA mixture and of 3.3 g of the WPH in the plasma EAA concentration changes and AUCs.

**Figure 2:**
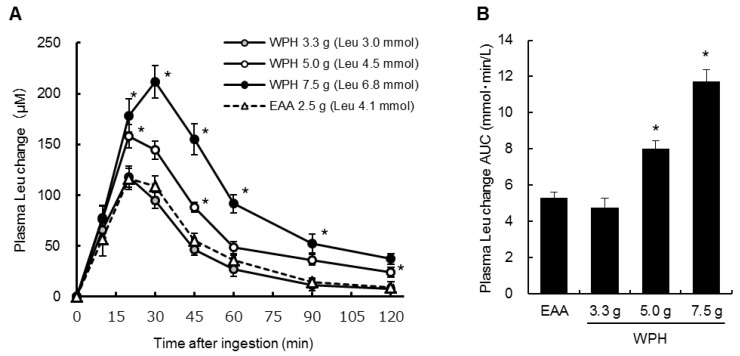
Plasma leucine concentration (**A**) changes from baseline and (**B**) AUCs. Values are mean ± standard error of the mean. * Significantly different from EAA, *p* < 0.05. Leu: leucine; AUC: area under the curve; WPH: whey protein hydrolysate; EAA: essential amino acids.

Figure 2A,B shows the changes from baseline and the AUCs of the plasma leucine concentrations. Significant treatment × time interactions were observed in the leucine concentration changes from baseline (*p* < 0.001). The leucine concentration changes at 20–90 min after ingestion of 7.5 g of the WPH and at 20, 45 and 120 min after ingestion of 5.0 g of the WPH were significantly higher than those after ingestion of the EAA mixture at the same time points. The plasma leucine concentration AUCs after ingestion of 5.0 and 7.5 g of the WPH were also significantly higher than that after ingestion of the EAA mixture. There were no significant differences between the ingestion of the EAA mixture and of 3.3 g of the WPH in the plasma leucine concentration changes and AUCs.

**Figure 3:**
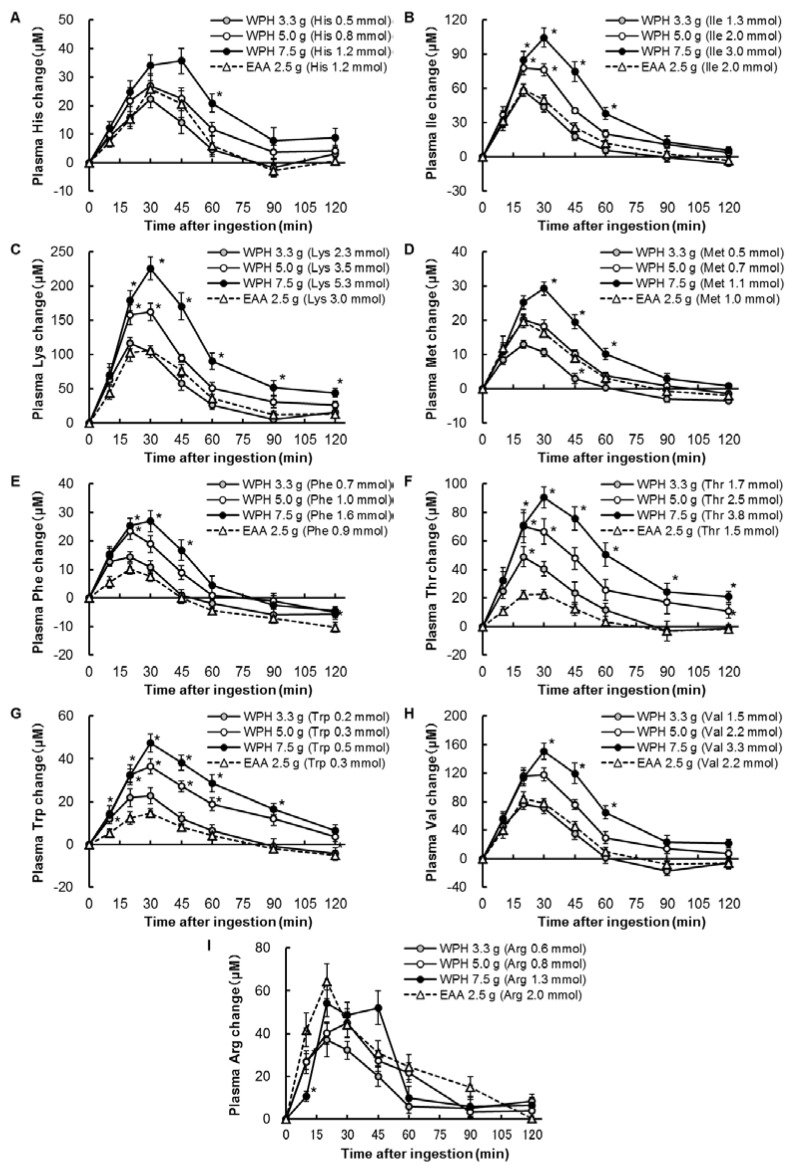
Plasma amino acids concentration changes from baseline. (**A**), His; (**B**), Ile; (**C**), Lys; (**D**), Met; (**E**), Phe; (**F**), Thr; (**G**), Trp; (**H**), Val; (**I**), Arg. Values are mean ± standard error of the mean. * Significantly different from EAA, *p* < 0.05. His: histidine; Ile: isoleucine; Lys: lysine; Met: methionine; Phe: phenylalanine; Thr: threonine; Trp: tryptophan; Val: valine; Arg: arginine; WPH: whey protein hydrolysate; EAA: essential amino acids.

The concentration changes of individual EAA (other than leucine) from baseline are presented in Figure 3. Significant treatment × time interactions were observed in all of the individual EAA analysis (*p* < 0.05).

There were no significant effects of subject or sequence in plasma EAA and all of the individual EAA concentration changes. Only in plasma histidine concentration change, there was a significant effect of period (*p* < 0.05).

There were significant effects of subject in plasma EAA, leucine, isoleucine, lysine, methionine, tryptophan, valine and arginine changes at 10 min after ingestion and of period in plasma EAA, histidine, isoleucine, lysine, phenylalanine and threonine changes at 90 min after ingestion (*p* < 0.05). There were also significant effects of period in plasma histidine changes at 10 and 45 min after ingestion (*p* < 0.05), and there were no significant effects of sequence in plasma EAA and all of the individual EAA concentration changes.

### 3.3. Plasma Insulin, Glucose and IGF-1

**Figure 4:**
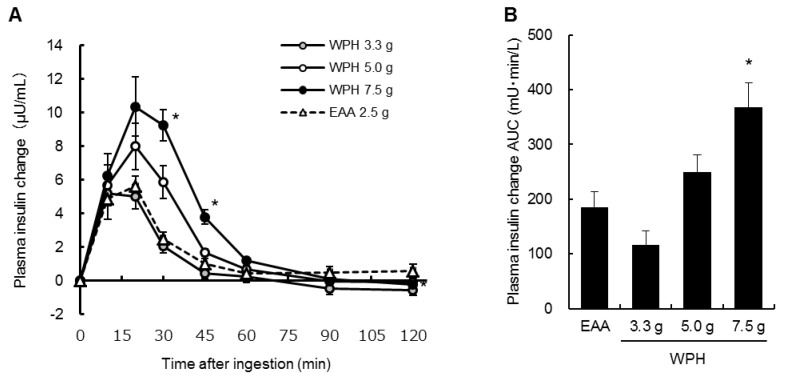
Plasma insulin concentration (**A**) changes from baseline and (**B**) AUCs. Values are mean ± standard error of the mean. * Significantly different from EAA, *p* < 0.05. AUC: area under the curve; WPH: whey protein hydrolysate; EAA: essential amino acids.

Figure 4A,B show the changes from baseline and the AUCs of the plasma insulin concentrations. Significant treatment × time interactions were observed in the insulin concentration changes from baseline (*p* < 0.001). The insulin concentration changes at 30 and 45 min after ingestion of 7.5 g of the WPH were significantly higher than those of the EAA mixture at the same time points. The plasma insulin concentration AUCs after ingestion of 7.5 g of the WPH were also significantly higher than that of the EAA mixture. There were no significant differences between the ingestion of the EAA mixture and of 3.3 and 5.0 g of the WPH in the plasma insulin concentration changes and AUCs.

**Figure 5:**
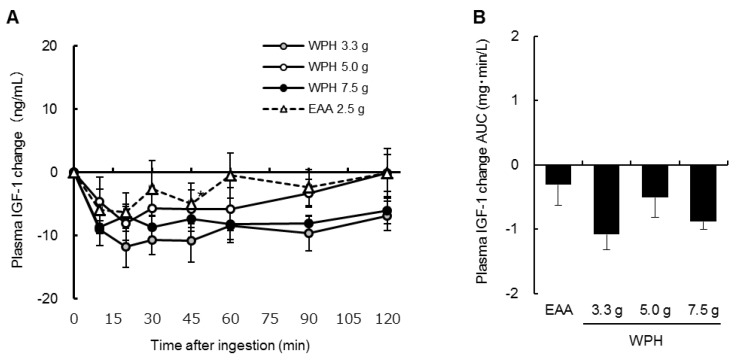
Plasma IGF-1 concentration (**A**) changes from baseline and (**B**) AUCs. Values are mean ± standard error of the mean. AUC: area under the curve; WPH: whey protein hydrolysate; EAA: essential amino acids.

Figure 5A,B show the changes from baseline and the AUCs of the plasma IGF-1 concentrations, and Table 3 shows the changes from baseline of the plasma glucose concentrations. In the plasma glucose and IGF-1 changes, there were significant main effects of time (*p* < 0.001), but there were no significant interactions or main effects of treatment. There were no significant differences between the ingestion of the EAA mixture and of each dose of the WPH in the plasma IGF-1 AUCs.

There were significant effects of subject in plasma insulin concentration changes at 10 min after ingestion. There were no significant effects of period or sequence in plasma insulin, IGF-1 and glucose concentration changes.

## 4. Discussion

The main purpose of this study was to investigate the effects of ingestion of WPH on postprandial aminoacidemia, especially plasma leucine appearance, compared to that of an amino acid mixture. We compared 3 doses (7.5, 5.0 and 3.3 g of protein) of the WPH to 2.5 g of the EAA mixture as the control, and we found that ingestion of 3.3 g of the WPH, which contained lower amounts of EAA and leucine compared with the EAA mixture, induced plasma EAA and leucine levels similar to that of the EAA mixture. Furthermore, even though 5.0 gm of the WPH and 2.5 g of the EAA mixture contained similar amounts of EAA and leucine, ingestion of 5.0 g of the WPH caused higher plasma EAA and leucine levels compared to the EAA mixture. In plasma histidine, isoleucine, methionine, phenylalanine, threonine, tryptophan and valine levels, ingestion of the WPH also induced higher aminoacidemia compared to the EAA mixture when those contained similar amounts of the amino acid. These results suggest that the WPH can induce a greater degree of aminoacidemia compared with a free amino acid mixture when those preparations are ingested orally. Although it is well known that free amino acids are absorbed rapidly and can induce rapid postprandial aminoacidemia, to our knowledge it is the first study that indicated that oral ingestion of protein hydrolysate may induce higher plasma amino acid levels compared with that of a free amino acid mixture.

Protein hydrolysis is the cleavage of peptide bonds, which breaks proteins down to peptides of different sizes and to free amino acids. The degree of this process can be defined by a global value known as degree of hydrolysis (DH) [20]. However, 2 protein hydrolysates with a similar DH may still differ substantially from each other. For example, one may contain mainly larger oligopeptides and free amino acids while the other contains mainly di-, tri- and tetrapeptides and fewer free amino acids. The WPH matches the latter, because it contains a low proportion of free amino acids (less than 1% of the WPH), and its average peptide chain length is 3.50. Amino acids from dietary protein are known to be absorbed not only by free amino acid uptake through amino acid transporters but also by di- and tripeptide uptake through peptide transporters [21]. Furthermore, in previous studies, di- and tripeptides were usually absorbed faster than free amino acid mixtures, which were all delivered by perfusion of the small intestine [22,23]. Those studies may explain how, in our study, WPH ingestion was followed by a higher level of aminoacidemia compared with the EAA mixture: the WPH may have been absorbed in the form of di- and tripeptides as well as free amino acids, while the EAA mixture was absorbed only in the form of free amino acids.

Protein absorption rates are strongly associated with postprandial plasma amino acid response rates. However, other amino acids besides those from dietary sources can affect the amino acid pool in the plasma. Whole body protein kinetics are complex: the supply of amino acids from the splanchnic bed, which includes the liver, stomach, intestines, pancreas, and spleen, is also associated with the amino acid pool in the plasma [24]. Furthermore, splanchnic bed tissue has been shown to extract nearly 60% of dietary nitrogen [25], because the protein synthesis rate of the splanchnic bed is higher compared to that of the skeletal muscle [24]. Therefore, the greater increase of plasma amino acids after WPH ingestion may have resulted from not only its faster absorption but also from an increased amino acid supply from the splanchnic bed or a decrease of dietary amino acid uptake by the splanchnic bed. Manninen [26] also suggests that rapid absorption of amino acids appears to decrease the splanchnic extraction of amino acids and therefore increases the magnitude of the acute increase of amino acids in the plasma. Further studies using stable isotope tracer techniques [24] are needed to verify this hypothesis.

The results in this study do not necessarily apply to other WPHs. Although Morifuji et al. [12] showed that the ingestion of WPH was associated with a greater increase of plasma amino acids compared with intact whey protein, Power et al. [27] showed that the postprandial rate of appearance of branched chain amino acids was not significantly different between WPH and intact whey protein. Additionally, although Grimble et al. [28] showed that an egg protein hydrolysate containing mostly di- and tripeptides was more rapidly absorbed than hydrolysates containing longer peptides, a more recent study by Farup et al. [29] used 3 different WPH with varying degrees of hydrolysis; and Farup et al. did not provide evidence that the degree of whey protein hydrolysis is a strong determinant for the postprandial amino acid appearance rate in plasma. It may not be sufficient merely to hydrolyze protein: specific hydrolysis conditions may be needed to affect the absorption kinetics, but such conditions are not yet known.

The sub-purpose of this study was to estimate the minimum effective dose of the WPH for stimulating MPS in terms of postprandial aminoacidemia. It is well known that essential amino acids are primarily responsible for the amino acid-induced stimulation of muscle protein anabolism [30], and leucine in particular plays an important role in the activation of MPS [31,32]. Elevation of plasma leucine levels is associated with MPS stimulation: the ‘leucine trigger’ hypothesis states that there may be a threshold level of plasma leucine to trigger MPS [33]. In this study, we used 2.5 g of the EAA mixture as the control, as it would cause the minimum required aminoacidemia level for increasing postprandial MPS [16]; and we found that ingestion of 3.3 g of the WPH was associated with a similar level of leucinemia compared with the EAA mixture. Insulin is another positive regulator of muscle protein metabolism [34], and the EAA mixture and the 3.3 g of the WPH were associated with similar plasma insulin responses. Therefore, we suggest ingestion of more than 3.3 g of the WPH is a proper design for further human studies to investigate the effects of the WPH on muscle protein metabolism. The dose is very low, because to our knowledge the lowest dose of intact whey protein associated with increased MPS in previous studies is 6.25 g [35].

Some limitations exist in this study. First, there might have been some problems with blood sample collecting or analysis at 90 min after ingestion because significant effects of period were found in plasma EAA and some amino acid concentration changes at 90 min after ingestion. Second, none of the treatments are matched for the EAA contents, and the difference of the EAA contents may affect the postprandial aminoacidemia. Moreover, other than EAA, WPH contains all the non-EAA that are not included in the EAA mixture. These non-EAA may positively affect the absorption and transport of the EAA in WPH. Further studies are needed to compare the WPH to free AA under the condition that both contain the same amino acids.

## 5. Conclusions

In conclusion, the WPH was associated with greater postprandial elevation of amino acids, including leucine, in plasma compared to a free amino acid mixture in young men when both were ingested orally. The findings in this study support a new nutrition strategy for improving postprandial plasma leucine and EAA availability, which impact muscle protein metabolism.

## Figures and Tables

**Table 1:** The Latin square design for four treatments ^1^.

Sequence	*n*	Period			
		1	2	3	4
1	3	A	B	C	D
2	3	B	D	A	C
3	3	C	A	D	B
4	3	D	C	B	A

^1^ A, 7.5 g of WPH; B, 5.0 g of WPH; C, 3.3 g of WPH; D, 2.5 g of the EAA mixture; WPH, whey protein hydrolysate; EAA, essential amino acid.

**Table 2:** The essential amino acid content and other nutrients of the test solutions ^1^.

	WPH			EAA
Protein	3.3 g	5.0 g	7.5 g	2.5 g
	Amino acid content (mmol)			
His	0.5	0.8	1.2	1.2
Ile	1.3	2.0	3.0	2.0
Leu	3.0	4.5	6.8	4.1
Lys	2.3	3.5	5.3	3.0
Met	0.5	0.7	1.1	1.0
Phe	0.7	1.0	1.6	0.9
Thr	1.7	2.5	3.8	1.5
Trp	0.2	0.3	0.5	0.3
Val	1.5	2.2	3.3	2.2
Arg	0.6	0.8	1.3	2.0
EAA	12.3	18.4	27.8	18.2
	Other nutrients (g)			
Carbohydrates	0.5	0.8	1.1	0.0
Fat	0.0	0.0	0.0	0.0

^1^ WPH, whey protein hydrolysate; EAA, essential amino acid.

**Table 3:** The changes from baseline of plasma glucose concentrations ^1^.

	Time after Ingestion						
	10 min	20 min	30 min	45 min	60 min	90 min	120 min
ΔGlucose (mg/dL)							
WPH 7.5 g	0.9 ± 0.7	2.9 ± 1.3	1.5 ± 1.1	−2.2 ± 1.9	−2.5 ± 1.0	−0.4 ± 1.2	0.9 ± 1.0
WPH 5.0 g	1.8 ± 1.0	1.9 ± 1.4	1.7 ± 1.2	−0.7 ± 1.8	−0.4 ± 1.1	0.1 ± 1.0	0.4 ± 1.0
WPH 3.3 g	1.2 ± 0.8	0.3 ± 1.0	−1.2 ± 1.4	−2.3 ± 1.1	−1.5 ± 1.1	−1.1 ± 0.8	−1.8 ± 0.8
EAA	2.5 ± 1.0	0.5 ± 1.2	−0.3 ± 1.1	−0.4 ± 1.2	−0.5 ± 0.9	−0.5 ± 0.8	−0.1 ± 1.2

^1^ Values are mean ± standard error of the mean. WPH: whey protein hydrolysate; EAA: essential amino acids.
